# Lymph node ratio predicts overall survival in patients with stage II non-small cell lung cancer: a population-based SEER analysis

**DOI:** 10.1007/s12672-022-00542-w

**Published:** 2022-08-18

**Authors:** Nan Feng, Bo Wu, Xiang Zhang, Jianhui Chen, Zhongtian Xiang, Yiping Wei, Wenxiong Zhang

**Affiliations:** Department of Thoracic Surgery, The Second Affiliated Hospital of Nanchang University, 1 Minde Road, Nanchang, 330006 China

**Keywords:** Non-small cell lung cancer, Stage II, Lymph node ratio, Survival, SEER

## Abstract

**Background:**

In non-small-cell lung cancer (NSCLC), there are many factors that affect prognosis, and the lymph node ratio (LNR) may play a significant role. Our study aimed to confirm the value of the LNR in the prognosis of patients with stage II NSCLC.

**Methods:**

Patient data were obtained from the Surveillance, Epidemiology and End Results (SEER) database. The classification for the LNR was best determined using the X-tile method. The correlation between the LNR and overall survival (OS) was validated after the Kaplan–Meier analysis was performed. To determine the correlation between the LNR and survival, stratification and the Cox regression analysis were used.

**Results:**

In our study, 14,183 stage II NSCLC patients were included. Among them, 8303 patients had N1 disease. According to the X-tile analysis, the optimal critical points for the LNR in N1 patients with NSCLC was 0.21 and 0.38. We categorized the cohorts as low (LNR-L ≤ 0.21; n = 5158, 62.1%), medium (0.21 < LNR-M ≤ 0.38; n = 1736, 20.9%), and high (LNR-H > 0.38; n = 1409, 17.0%). According to the Kaplan–Meier analysis, the patients with a high LNR were considerably worse than those with a medium or low LNR (P < 0.001), which was also proven by stratified and multivariate analyses. The value of the LNR was reflected in all the subgroup analyses, especially in patients ages < 60 years. The multivariate competing risks regression analysis revealed that younger age, female sex, T1 disease, adenocarcinoma and N0 disease was associated with a better prognosis after controlling for potential confounders (P < 0.001).

**Conclusions:**

For patients with stage II NSCLC, the LNR is valuable for assessing prognosis. A higher LNR indicates a worse prognosis.

## Introduction

The most common type of cancer is lung cancer, accounting for 12.3% of all cancer cases [1]. Non-small-cell lung cancer (NSCLC) accounts for the highest proportion of lung cancer cases, and the proportion of stage II patients was 9.8% [2]. The involvement of lymph nodes (LNs) in patients with NSCLC is already considered to be a prognostic factor [3]. For patients with primary NSCLC, accurately staging the status of the lymph node improves the patient’s prognosis and it also plays an important role in selecting specific treatment options for patients [4].

According to the tumor, node and metastasis (TNM) staging system, which no regional lymph nodes were involved and only ipsilateral lymph node involvement in the lungs, peri-bronchi, or hilar lymph nodes would be called N0 and N1. Patients with involvement of the ipsilateral mediastinal or sub-cardiac lymph nodes and involvement of the contralateral lymph nodes would be called N2 and N3 [5]. However, lymph node class is affected by retrieval and examination of the adequacy of lymph nodes, and the results obtained tend to vary. It may be considerable interference with the accurate staging of NSCLC. This in turn affects the prediction of the patient's prognosis. The number of positive LNs also can serve as an independent factor in assessing the prognosis of NSCLC patients, which has been validated in previous studies [6–8]. The quantity of sampled LNs is a limitation that affects the specific number of positive LNs because a single lymph node may be divided into multiple nodes due to manipulation in the sampling. This also hinders the application of the actual positive LN count as a prognostic factor.

In order to get the more accurate lymph node staging status. We can skip the above limitation by using the variable LNR, which is defined as the ratio obtained by dividing the number of positive LNs by the total number of LNs that were resected [9, 10]. The LNR has an important predictive role in patient prognosis and has been validated in colon, breast, gastric, esophageal and bladder cancers. This study aims to explore whether the LNR is helpful for predicting the prognosis of patients with stage II NSCLC and whether it can play an important role in the treatment plan [11–15].

## Materials and methods

### Patient data source

Detailed patient data were obtained through the Surveillance, Epidemiology and End Results (SEER) registry. All patients in the study were pathologically diagnosed with stage II NSCLC using the American Joint Committee on Cancer (AJCC) 8th edition diagnostic criteria. All patients underwent surgery between 2000 and 2018 [16]. In patients receiving preoperative radiation therapy, this could lead to a reduction in LN involvement, and patients with incomplete data including LN involvement, tumor expansion, and tumor size, were also excluded from the study.

### Data retrieval

After screening, we screened 14,183 patients from SEER* stat software version 8.4.0. These screened patients all included the following variables: number of positive lymph nodes, sex, histology, primary site, age, race, positive node (pN), positive tumor (pT), number of lymph nodes examined, duration of survival and survival status.

### Statistical analysis

The ratio obtained by dividing the number of positive LNs by the total number of resected LNs was used to determine the LNR of each patient included in the study [17]. Among stage II NSCLC patients, the principal result in this study was the evaluation of the effect of the LNR on survival and prognosis, and the secondary outcome was the influence of the prognosis-related factors.

The Kaplan–Meier method was used to determine survival trends in patients with stage II NSCLC, and a log rank test was used for comparisons [18]. According to the LNR, we divided patients in the N1 group into three groups: low, medium, and high. The cutoff points were calculated by X-tile, and the survival rate was estimated. After excluding other postprognostic factors that interfered, the Cox regression analysis was used to evaluate the effect of the LNR on survival [19].

## Results

### Population characteristics and demographics of the study

**Fig. 1:**
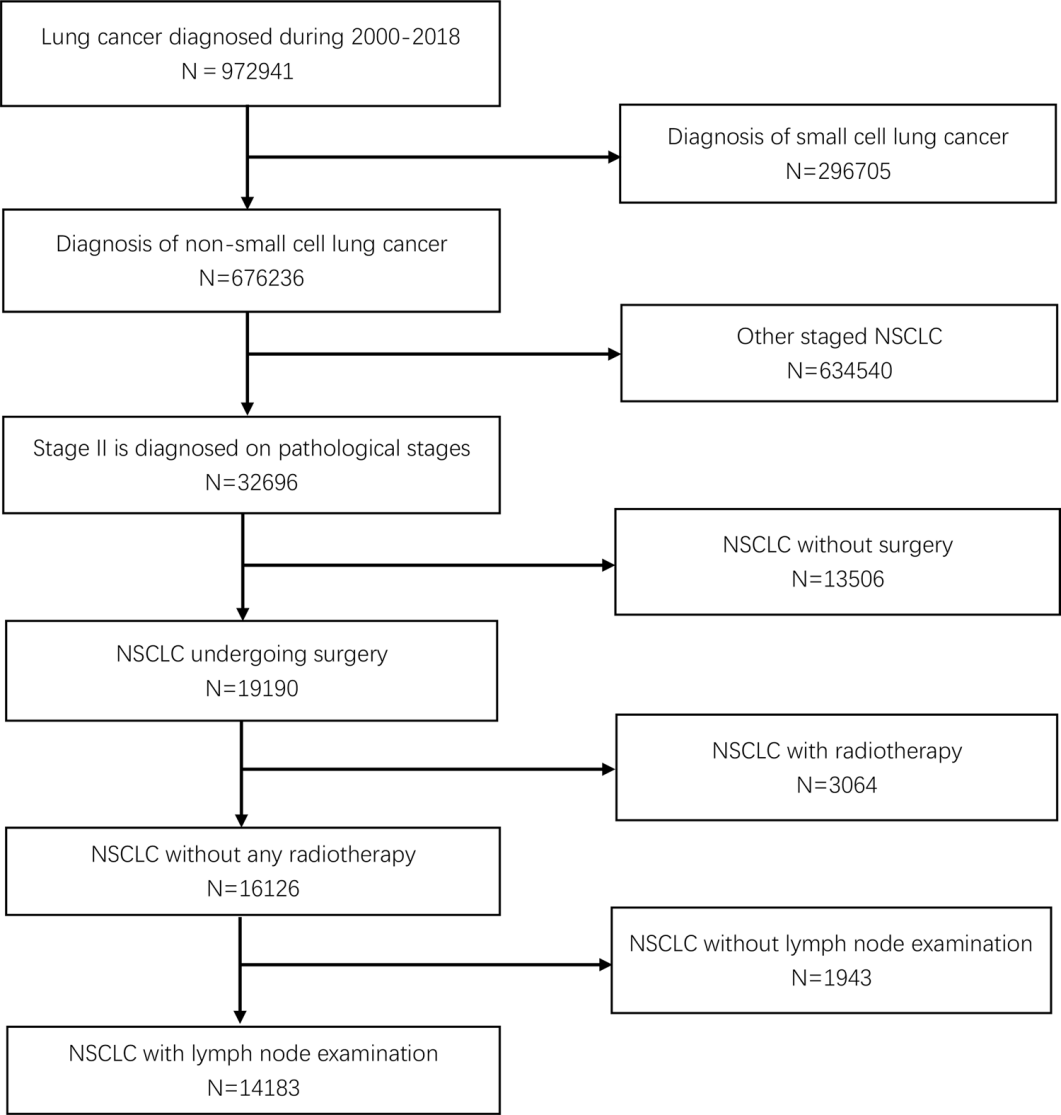
The research flow chart

The diagram of the research flow is shown in Fig. 1. We screened data from 2000 to 2018 and a total of 972,941 patients were diagnosed with lung cancer. Based on our screening criteria, 14,183 people with stage II NSCLC were matched in our study. In our population, the upper lobes are the most common primary site (56.1%); adenocarcinomas (56.7%) are the most prevalent histological subtypes, and more than half (54.1%) of cases affect males. In addition, 20.0%, 50.9% and 29.1% of the NSCLC patients had T1, T2 and T3 diseases. The description of the demographics and baseline characteristics of the patients is provided in Table 1.

**Table 1:** Baseline characteristics of patients with stage II non-small cell lung cancer

Characteristics	No. (%)
Sex	
Male	7667 (54.1)
Female	6516 (45.9)
Race	
Black	1295 (9.1)
White	11,981 (84.5)
Other	907 (6.4)
Age	
< 60	2766 (19.5)
60–75	7551 (53.2)
> 75	3866 (27.3)
T stage	
T1	2836 (20.0)
T2	7233 (50.9)
T3	4114 (29.1)
N stage	
N0	5880 (41.5)
N1	8303 (58.5)
Histology	
Adenocarcinoma	8041 (56.7)
Squamous cell carcinoma	4835 (34.1)
Other	1307 (9.2)
Primary site	
Upper lobe	7952 (56.1)
Lower lobe	4968 (35.0)
Main bronchus	142 (1.0)
Middle lobe	637 (4.5)
Other	484 (3.4)

*T* tumor; *N* node

### Factors influencing the prognosis of stage II NSCLC

**Fig. 2:**
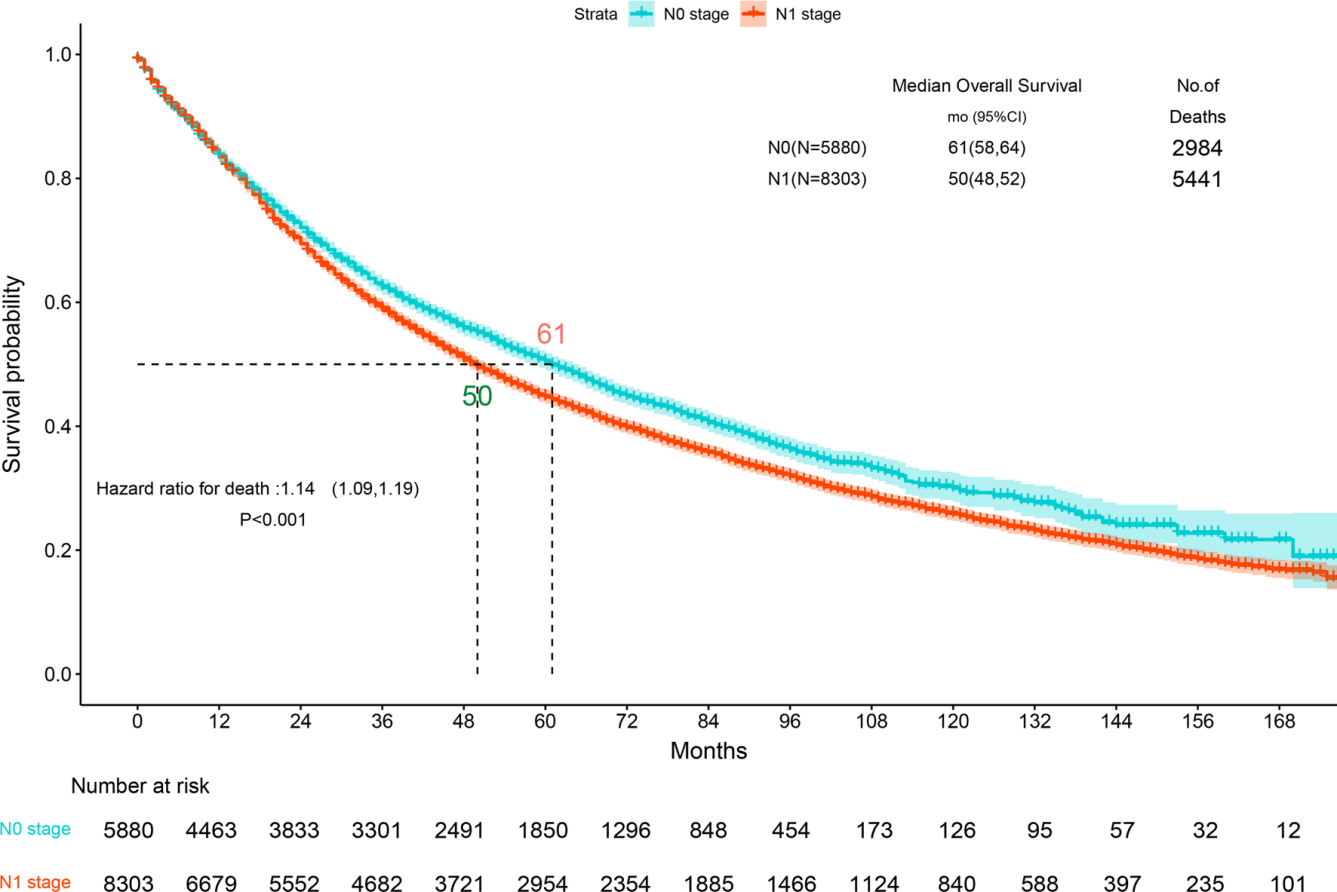
Kaplan–Meier survival curves for stage N0 patients versus stage N1 patients

**Fig. 3:**
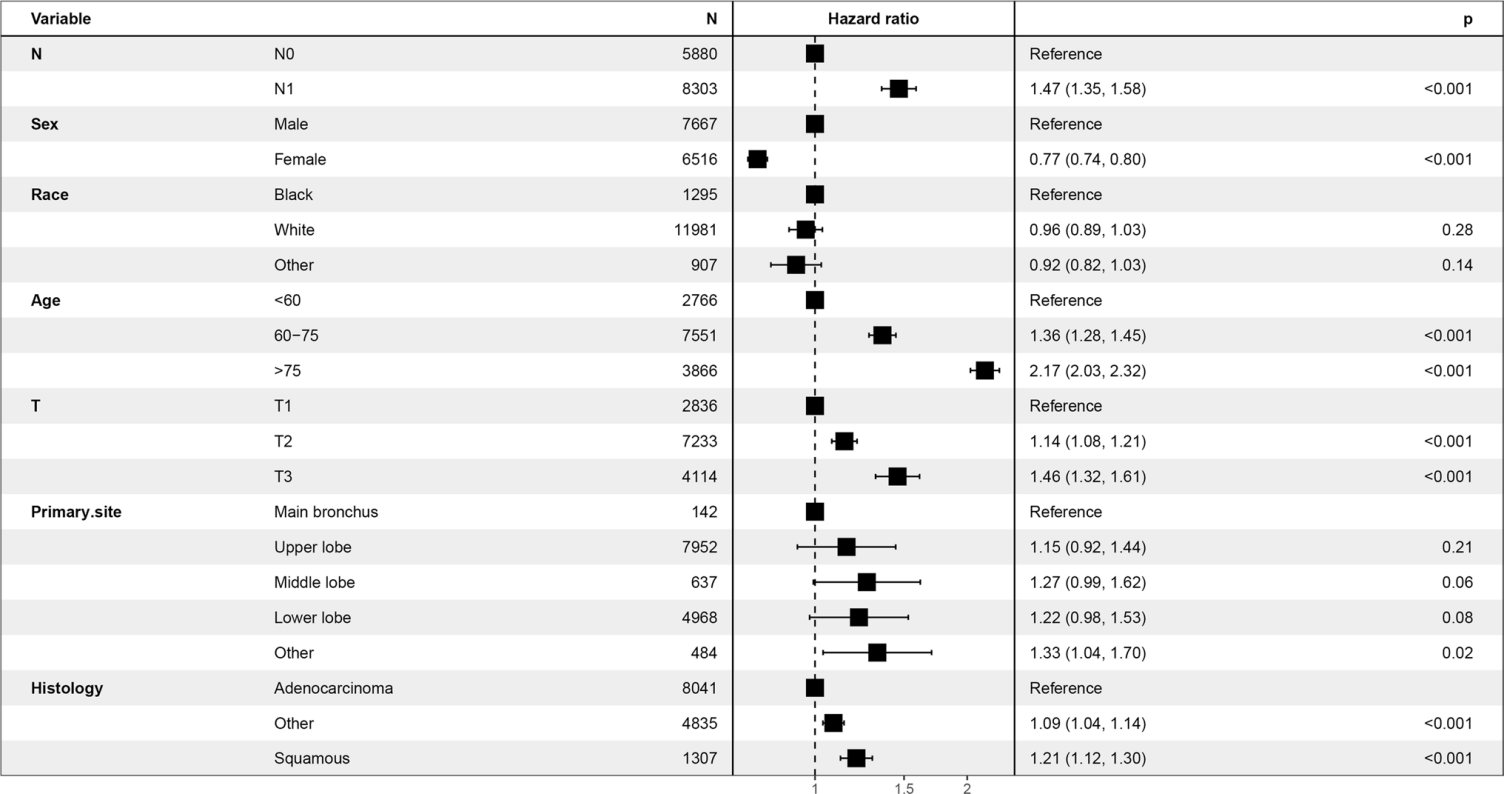
Forest plot demonstrating the results of multivariate Cox regression analysis of OS predicted by pN as a covariate

In the Kaplan–Meier analysis, the overall survival (OS) of the N0 group is better than that of the N1 group (P < 0.001, Fig. 2). In addition, using pN as a covariate, our multifactorial Cox regression analysis found that younger age, female sex, T1 disease, adenocarcinoma and an N0 status was associated with a better prognosis after controlling for potential confounders (P < 0.001, Fig. 3). Old age was an independent and significant predictor of OS (HR: 2.17, 95% CI 2.03–2.32; P < 0.001).

### Prognostic stratification of the N1 group according to LNR

**Fig. 4:**
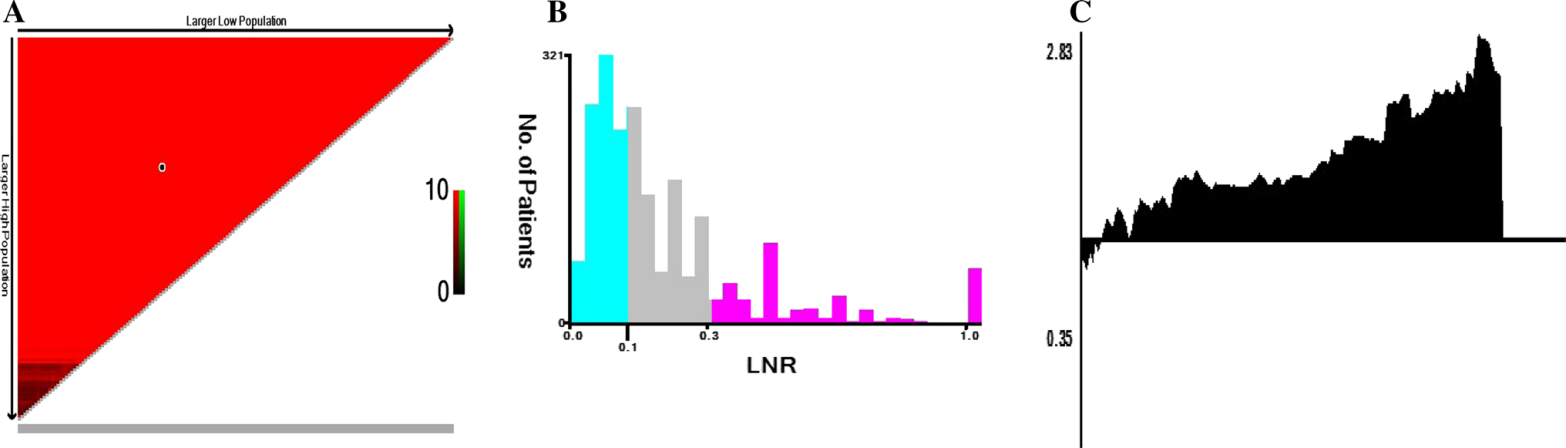
The best cut-off values for the lymph node ratio (LNR) by using X-tile. **A** The cut-point for the entire queue. **B** The histogram of the entire cohort. **C** The relative risk (RR) of the entire queue

**Fig. 5:**
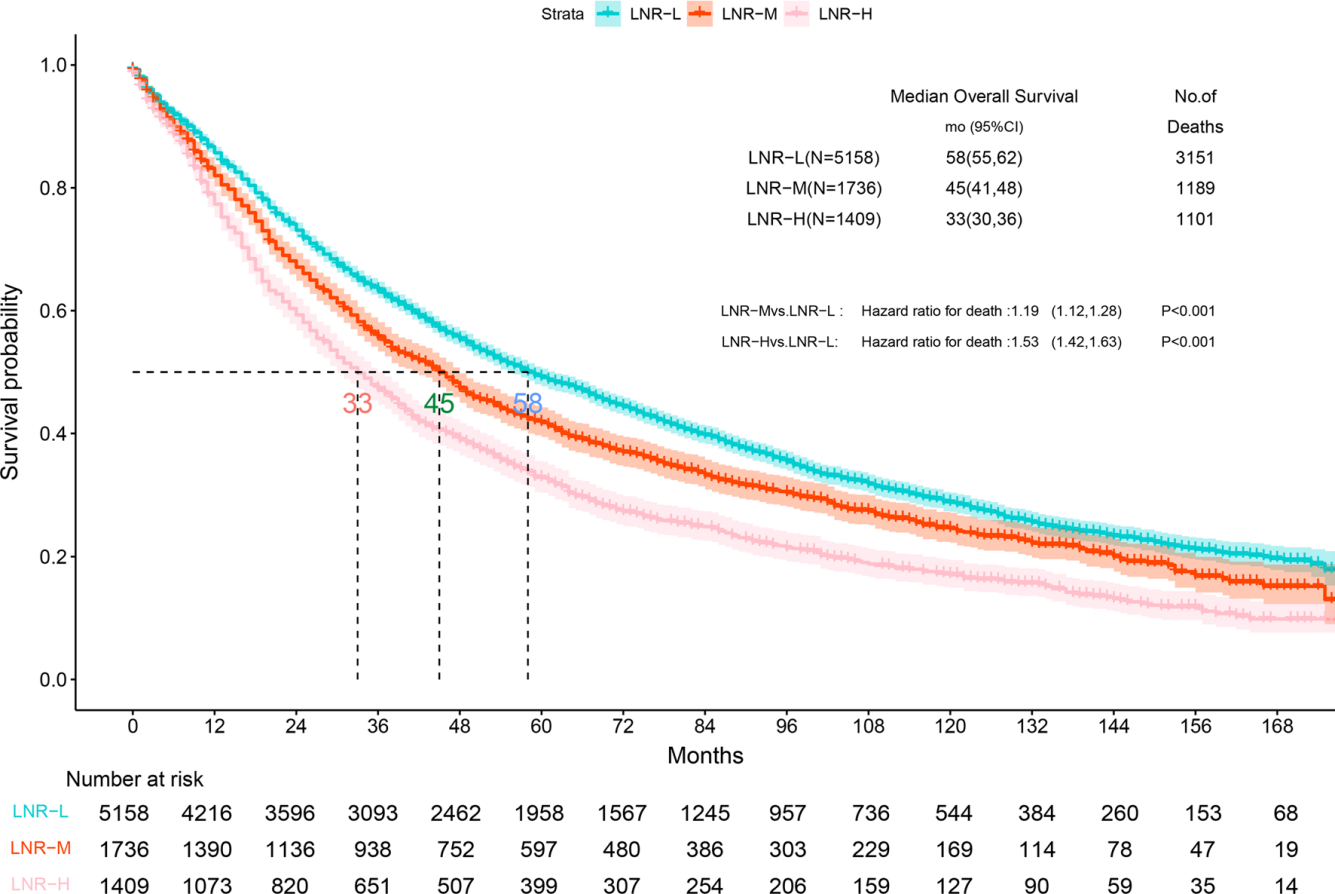
Kaplan–Meier survival curves for stage LNR-L patients versus stage LNR-M patients and stage LNR-H patients

**Fig. 6:**
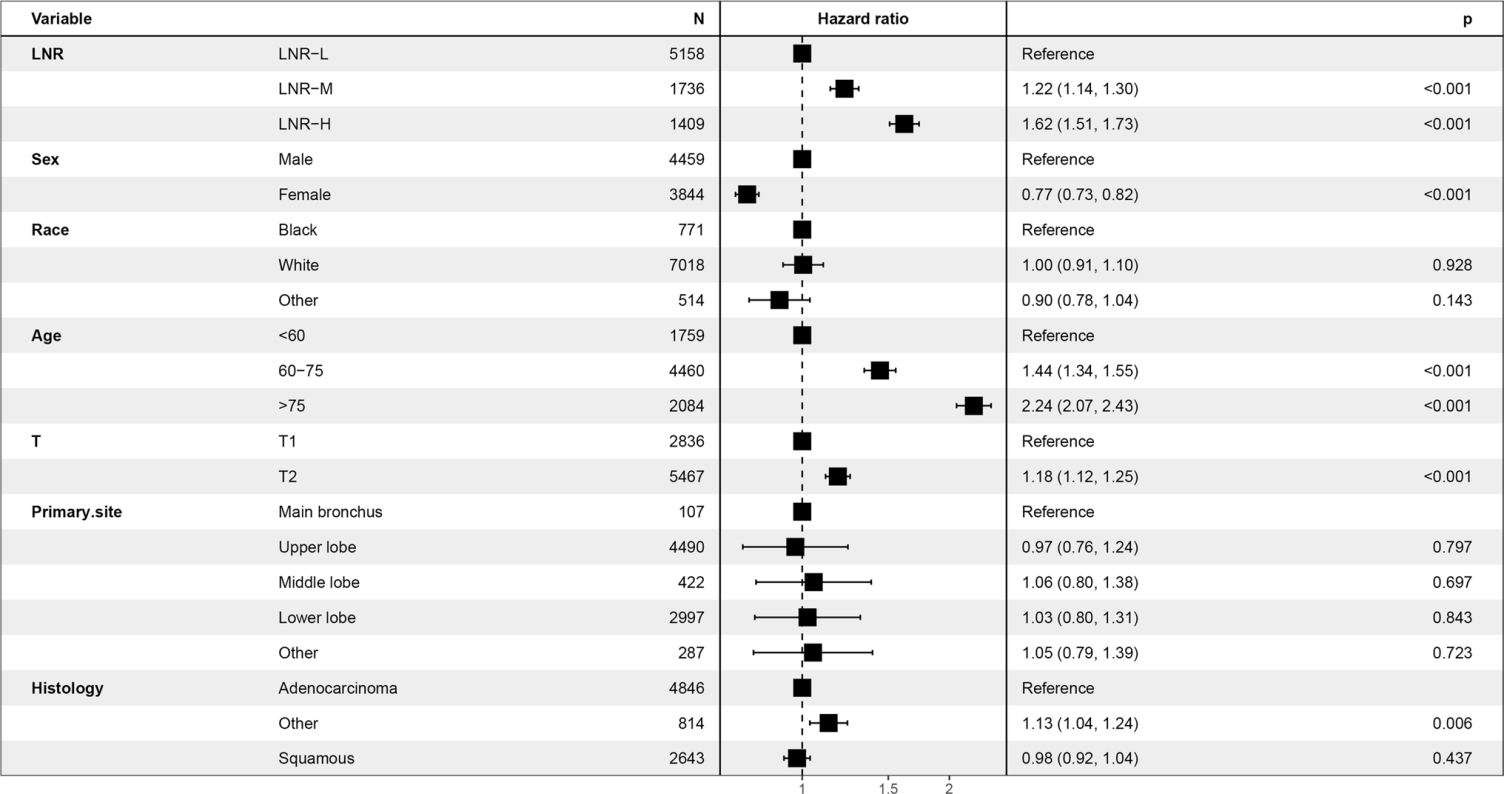
Forest plot demonstrating the results of multivariate Cox regression analysis of OS predicted by LNR as a covariate

For assessing prognosis in patients with stage II NSCLC, the most convenient and recognized indicator remains the TNM staging system. However, N-stage is affected by several factors, such as T-stage, age, pathologist judgment, tumor location and degree of lymphadenectomy. Therefore, we used the LNR to conduct a stratified study of prognosis. The optimal LNR thresholds for patients with N1 NSCLC are 0.21 and 0.38, which we obtained via X-tile calculation. We categorized the cohorts as low (LNR-L ≤ 0.21; n = 5158, 62.1%), medium (0.21 < LNR-M ≤ 0.38; n = 1736, 20.9%), and high (LNR-H > 0.38; n = 1409, 17.0%) (Fig. 4). Furthermore, the Kaplan–Meier analysis revealed that patients with a low LNR had significantly longer survival times than patients in the other subgroups (P < 0.001, Fig. 5). Using LNR as a covariate, our multifactorial Cox regression analysis found that among the independent adverse predictors of survival, LNR was a significant factor (LNR-H vs. LNR-L, HR: 1.62, 95% CI 1.51–1.73, P < 0.001, Fig. 6).

### Subgroup outcomes of different according to factors

**Fig. 7:**
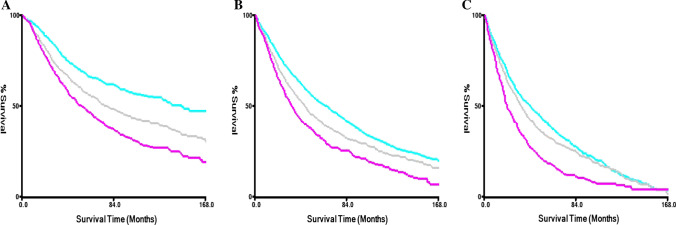
Kaplan–Meier survival curves is drawn for the age subpopulation stratified by the LNR. **A** The stage < 60. **B** The stage 60–75. **C** The stage > 75

**Fig. 8:**
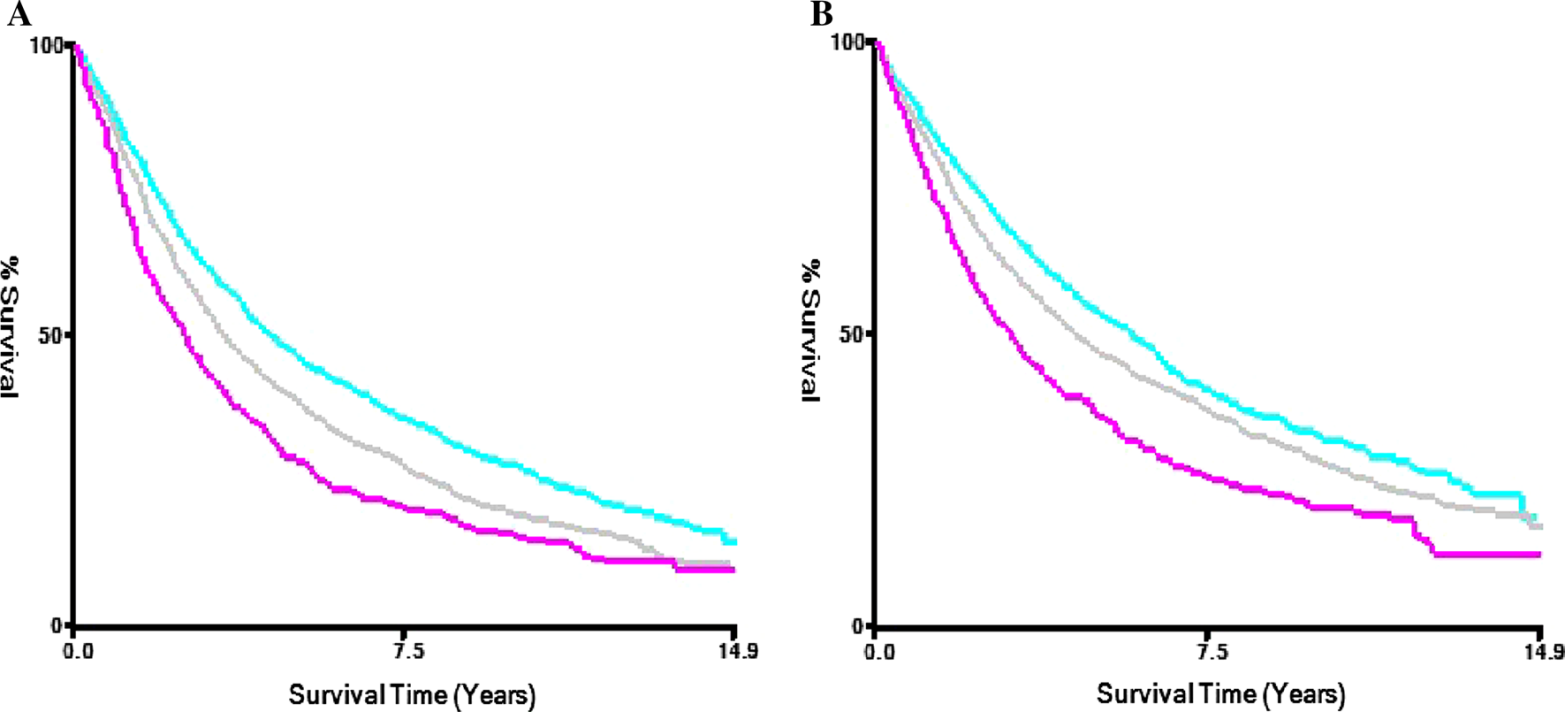
Kaplan–Meier survival curves is drawn for the number of lymph nodes subpopulation stratified by the LNR. **A** The number < 10. **B** The number > 10

The intercept point of the LNR is different for different factors, and the specific values and associated risk values are shown in Table 2. In our study, we found that regarding sex, age, T stage, race, histological type and primary site, survival was associated with a low LNR. The predictive value of age is particularly pronounced in patients who are younger than 60 years. (LNR-H vs. LNR-L, HR: 2.04, 95% CI 1.71–2.44, P < 0.001). Patients younger than 60 years of age and with a high LNR had a significantly shorter survival time, which was verified in the Kaplan–Meier analysis (Fig. 7A–C). This result might be more beneficial for younger patients with stage II NSCLC. We also divided the number of lymph nodes by a cut-off of 10, which is a relatively common quantity [20–22]. From the results we derived, we can see that the LNR grouping is a guide to patient prognosis, regardless of the total number of lymph node dissection (Fig. 8A, B).

**Table 2:** The univariate multi-subgroup Cox regression model of LNR

Subgroups	Cut-off points (low)	Cut-off points (high)	LNR-L	LNR-M		LNR-H	
			HR (95% CI)	HR (95% CI)	*P*	HR (95% CI)	*P*
Total	0.21	0.38	1 (Reference)	1.19 (1.12–1.28)	< 0.001	1.53 (1.42–1.63)	< 0.001
Sex							
Male	0.17	0.38	1 (Reference)	1.18 (1.09–1.28)	< 0.001	1.64 (1.49–1.80)	< 0.001
Female	0.13	0.29	1 (Reference)	1.12 (1.01–1.23)	0.02	1.50 (1.36–1.66)	< 0.001
Age							
< 60	0.1	0.29	1 (Reference)	1.50 (1.27–1.78)	< 0.001	2.04 (1.71–2.44)	< 0.001
60–75	0.21	0.46	1 (Reference)	1.26 (1.16–1.38)	< 0.001	1.64 (1.48–1.81)	< 0.001
> 75	0.21	0.38	1 (Reference)	1.12 (1.01–1.25)	0.04	1.66 (1.45–1.89)	< 0.001
T stage							
T1	0.21	0.38	1 (Reference)	1.13 (1.01–1.28)	0.04	1.62(1.44–1.82)	< 0.001
T2	0.15	0.31	1 (Reference)	1.15 (1.06–1.24)	< 0.001	1.49 (1.38–1.61)	< 0.001
Histological type							
Adenocarcinoma	0.21	0.38	1 (Reference)	1.23 (1.12–1.34)	< 0.001	1.60 (1.47–1.75)	< 0.001
Squamous cell carcinomas	0.06	0.31	1 (Reference)	1.37 (1.05–1.78)	0.02	1.78 (1.34–2.36)	< 0.001
Other	0.09	0.33	1 (Reference)	1.18 (1.05–1.32)	0.005	1.58 (1.36–1.84)	< 0.001
Race							
Black	0.1	0.3	1 (Reference)	1.41 (1.11–1.80)	0.004	1.74 (1.36–2.23)	< 0.001
White	0.21	0.38	1 (Reference)	1.19 (1.10–1.27)	< 0.001	1.55 (1.44–1.67)	< 0.001
Other	0.21	0.4	1 (Reference)	1.42 (1.09–1.86)	0.01	1.76(1.34–2.31)	< 0.001
Primary site							
Main bronchus	0.13	0.24	1 (Reference)	2.02 (1.12–3.64)	0.02	1.59 (0.88–2.86)	0.12
Upper lobe	0.11	0.38	1 (Reference)	1.12 (1.03–1.22)	0.008	1.49 (1.34–1.68)	< 0.001
Middle lobe	0.12	0.22	1 (Reference)	1.22 (0.88–1.70)	0.2	1.61 (1.22–2.12)	< 0.001
Lower lobe	0.14	0.29	1 (Reference)	1.16 (1.04–1.29)	0.007	1.69 (1.52–1.88)	< 0.001
Other	0.12	0.22	1 (Reference)	1.00 (0.68–1.48)	0.992	1.58 (1.17–2.15)	0.003
Number of lymph nodes							
< 10	0.25	0.56	1 (Reference)	1.26 (1.15–1.37)	< 0.001	1.58 (1.42–1.76)	< 0.001
> 10	0.07	0.31	1 (Reference)	1.16 (1.06–1.27)	< 0.001	1.57 (1.37–1.79)	< 0.001

*HR* hazard ratio; *LNR* lymph node ratio; *N* node; *P* P-value; *T* tumor

## Discussion

For patients with NSCLC, the number of LN resections is a major prognostic factor, as has been demonstrated in many studies [23]. In studies on N1 and N2 patients, it was found that metastasis of a greater number of LNs had a worse prognostic impact [24]. However, in their studies, the number of LNs that exist will be affected by many factors, which may interfere with the results. To avoid interference with these conditions, we used the LNR to assess patient prognosis. The LNR has an important predictive role in patient prognosis and has been validated in colon, esophageal, gastric, bladder, and breast cancers [11–15]. However, in NSCLC, the role of LNR has not been proven. Li et al. found a positive correlation between lung nodal status and lung cancer prognosis in 301 patients with N1 and N2 diseases [25]. These results should be applied in the clinical setting and confirmed in an independent population. Patients with advanced lung cancer are more likely to die at competitive risk. Therefore, the importance of applying the LNR to assess prognosis in patients with early-stage lung cancer will become more important.

In our study, we selected 14,183 stage II NSCLC patients for a population-based analysis. According to this study, patients with stage II NSCLC have different prognoses depending on their LNR grade. The OS of patients with a high LNR was considerably worse than of those with a medium or low LNR (P < 0.001). In a previous study, in elderly N1 patients, the LNR was found to be an important factor in prognosis [26]. In our study, this conclusion was validated by LNR stratification in patients older than 75 years. In addition, our study found that, in patients younger than 60 years, the reduction in OS was more pronounced in the higher LNR group were more pronounced. This result might be more beneficial for younger patients with stage II NSCLC. In addition to age, we also verified the impact of different sexes, T-stage, races, histological types, and primary sites [27]. Our cut-off values for LNR were derived by X-tile stratification of N1 patients over the last 20 years obtained from SEER database. The cutoff point of LNR will change under different restrictions. But among the overall population, we have demonstrated by Cox analysis that it is an independent prognostic factor. So, we can conclude that LNR is a valid predictor of prognosis for stage II NSCLC in clinical practice [28].

LNR also has an important role in the selection of specific treatment options for patients. The LNR informs doctors of the prognosis of survival and it will improve the accuracy of staging and help doctors provide adjuvant treatment to patients with NSCLC [29, 30]. Because patients with a higher LNR have a greater risk of recurrence, it is important to consider aggressive postoperative treatment. At present, the treatment guidelines for stage II NSCLC with lymph node metastasis can be considered. It is recommended to perform surgical resection first and then adjuvant platinum chemotherapy [29, 31]. However, adjuvant chemotherapy has been found to have more toxic and long-term adverse effects in patients of advanced age with multiple underlying disease comorbidities, or other specific diseases [32]. It is difficult for these patients to tolerate these adverse effects before receiving the benefits of adjuvant chemotherapy. Therefore, the decision to use adjuvant chemotherapy has become particularly important. Previously, N stage patients were assessed to determine whether adjuvant therapy was needed because adjuvant therapy is more affected by lymph node sampling. However, the use of TNM staging to assess the lymph node staging status was subject to many interferences, and thus accurate staging results were not obtained. In this study, we considered using LNR staging as an alternative to N staging to select a treatment option. We study corollary: in patients with stage II NSCLC, if the LNR < 0.21, doctors can consider retaining adjuvant treatment for these patients. After our verification, in early NSCLC, the LNR is valuable in helping physicians develop specific treatment plans and long-term treatment plans for patients. The survival prognosis of patients in low LNR group seemed to be satisfactory, which is similar to patients in stage I. Therefore, it is debatable whether these patients need adjuvant treatment (immunotherapy, targeted therapy, chemotherapy, etc.) after surgery or not. In patients with lung cancer complicated with LN, postoperative radiotherapy (PORT) is still controversial. LNR may also have a considerable role in the use of radiotherapy in the future [33]. The PORT meta-analysis showed no evidence that PORT has obvious benefits [34]. Some studies have even shown that PORT results in a worse prognosis in patients who underwent complete resection for NSCLC [35]. The LNR should be well controlled in future studies that evaluate the effects of radiotherapy, because the LNR, a potential confounding factor, can have an impact on the survival assessment of patients who received radiotherapy.

In our study, the cohort population that was studied was patients with stage II NSCLC, and the prognostic factors in lung cancer patients at an early stage were discussed. According to previous literature, studies have been conducted in all NSCLC patients without indicating whether there is a difference in specificity in early-stage patients, and our study compensates for this limitation. Furthermore, the current research has some limitations. The main sources of bias in this study are its retrospective design. The surgeries were performed by multiple surgeons, there was no analysis of disease-free survival, and the groups included were heterogeneous in this study. Due to the lack of preoperative clinical TNM staging in the SEER database, we are unable to accurately stage preoperative LNR to guide practice [36]. Meanwhile, the lack of clinical data to verify our results may reduce the reliability of the conclusions of the study.

## Conclusion

Through our study of patients with stage II NSCLC, the LNR was found to be a valuable factor for assessing prognosis. A higher LNR indicates a worse prognosis. The assessed value of LNR was reflected in all the subgroup analyses, especially for patients ages < 60 years. Patients with a higher LNR have worse outcomes, so more aggressive treatment should be considered, and recurrence monitoring should be improved. Our results provide evidence that supports the inclusion of the LNR in the lung cancer staging system in the future. However, due to the lack of clinical data for verification, more large-scale, high-quality RCTs are required to verify this conclusion.

## Data Availability

The data sets used and/or analysed during the current study are available from the corresponding author on reasonable request.

## Abbreviations

AJCC: American Joint Committee on Cancer
HR: Hazard ratio
KM: Kaplan–Meier
LNR: Lymphnode ratio
LN: Lymph node
NSCLC: Non-small cell lung cancer
OS: Overall survival
pN: Positive node category
pT: Positive tumor
PORT: Postoperative radiotherapy
RR: Relative risk
SEER: Surveillance epidemiology and end results
SCLC: Small cell lung cancer
TNM: Tumor node metastasis
